# Audiovestibular Symptoms in Osteoporotic Postmenopausal Dominican Women

**DOI:** 10.7759/cureus.25300

**Published:** 2022-05-24

**Authors:** Ricardo Acra-Tolari, Dolores Mejía, Marcos Mirambeaux, Alicia Germán Dihmes

**Affiliations:** 1 Otolaryngology-Head and Neck Surgery, General Hospital Plaza de la Salud, Santo Domingo, DOM; 2 Endocrinology, Diabetes and Metabolism, General Hospital Plaza de la Salud, Santo Domingo, DOM; 3 Research, General Hospital Plaza de la Salud, Santo Domingo, DOM

**Keywords:** postmenopausal, tinnitus, hearing impairment, vertigo, audiovestibular, osteoporosis

## Abstract

Introduction: Previous publications have suggested a link between decreased bone mineral density (BMD) and audiovestibular symptoms. Being the first study, to our knowledge, in the Caribbean region, we aimed to determine the relationship between hearing impairment, vertigo, and tinnitus with BMD in a Dominican postmenopausal population diagnosed with osteoporosis.

Methods: A retrospective, cross-sectional, single-center study was conducted. Patients were enrolled using a previous database of Dominican postmenopausal women from 2008 to 2015, including a total of 101 participants. Patients were questioned for hearing difficulty, tinnitus, and vertigo. Body mass index (BMI), Bone Mineral Density (BMD) using T-scores, FRAX score, serum levels of 25-(OH) vitamin D, corrected calcium, phosphate, albumin, alkaline phosphatase, and smoking history were assessed. Statistical analysis was conducted using Chi-square, Mann-Whitney U test, and Odds Ratio.

Results: Median age was 74 years with an interquartile range (IQR) of 12. Fifty-six patients had osteoporosis and 42 osteopenia. Women with osteoporosis diagnosed using a femoral neck T-score demonstrated a greater level of association as well as higher odds of developing vertigo and hearing difficulty than those diagnosed using a hip T-score.

Conclusion: Special attention to the otologic evaluation of these patients as well as increasing the referral to a specialized consult is suggested. Additional multicenter prospective national studies are needed.

## Introduction

Presbycusis, or age-related hearing loss, is a common affliction of the elderly. It typically begins around the age of 60, but certain stressors have been shown to accelerate its rate of decline. Initially believed to be associated with degenerative changes in the peripheral auditory system and plasticity in central neural processing, presbycusis is now recognized as a complex condition involving genetic predisposition, anatomical changes, noise exposure, hormones, ototoxic medications, head trauma, ear-related conditions, and some systemic diseases [1-4]. Previous studies proposed the association between osteoporosis and hearing loss, although conflicting results have been stated [4]. 

The World Health Organization (WHO) predicts that by 2025, almost 500 million people aged 60 and older will have substantial age-related hearing loss [1]. Its impact has been demonstrated to largely affect low and middle-income countries, which have greater age-standardized rates of moderate-to-complete hearing loss than high-income countries. In addition, the disability burden attributable to hearing loss is concentrated in countries with limited access to health care, where hearing aid coverage is low and patients are least likely to obtain the care they require [5].

Osteoporosis is a systemic skeletal disease characterized by low bone mass and microarchitecture deterioration of bone tissue, resulting in increased fracture risk [6]. It is also the most prevalent illness in postmenopausal women, primarily owing to estrogen's crucial role in maintaining bone health [7]. Reduced bone mineral density (BMD) has been associated with demineralization of the temporal bones, specifically their petrous portion, which contains the components of the inner ear. This might partially explain the association between hearing loss and osteoporosis. Other studies have linked impaired calcium metabolism to benign paroxysmal positional vertigo (BPPV), which may be due to the aforementioned, but also by a hormonal component in postmenopausal women. Especially as estrogen has been suggested to influence both hearing and vestibular functions, playing a critical role in the maintenance of otoconia [4,7-9].

This has drawn our attention towards the development of strategies that could directly impact the improvement of quality of life among these patients. Being the first study, to our knowledge, in the Caribbean region to evaluate this relation, we aimed to determine the association between audiovestibular symptoms and osteoporosis in a postmenopausal population and assess the odds of developing these symptoms in patients with osteoporosis.

## Materials and methods

A retrospective, cross-sectional, single-center study was conducted. Patients were enrolled using a previous database, from 2008 to 2015, in which Dominican postmenopausal women were thoroughly examined, including their audiological and vestibular sphere. The current study enrolled a total of 101 participants. Patients were divided into three groups, women with a history of vertigo, women with a history of hearing difficulty, and women with a history of tinnitus. 

Audiovestibular symptoms were assessed using self-reported qualitative data. Patients were questioned for hearing difficulty, tinnitus, and vertigo, as well as if they had a history of recurrent otitis media, impacted cerumen, Menier's disease, otosclerosis, a previous tympanoplasty, cochlear implant, myringotomy, and family history of hearing impairment. In addition, their smoking history was assessed.

Body mass index (BMI) was calculated by dividing weight by the square of height (kg/m²). The femoral neck and total hip bone mineral density (g/cm²) were measured using bone density scans (DXA). The T-score is defined as the difference between a measured bone density and the expected young normal value divided by the population standard deviation (SD) derived from manufacturer-supplied guidelines. The WHO T-score criteria were used to diagnose osteopenia or osteoporosis; T-score ≥ 1 was considered normal; T-score < -1 was considered low bone mass (osteopenia); and T-score ≤ -2.5 osteoporosis [10]. The Fracture Risk Assessment Tool (FRAX) score, a web-based algorithm designed to calculate the 10-year probability of major osteoporosis-related fractures [11], was also determined. Serum levels of 25-(OH) vitamin D, corrected calcium, phosphate, albumin, and alkaline phosphatase were tested using automated standard laboratory procedures. 

Statistical analyses were performed using JASP version 0.13.1 for Mac, data were tabulated in spreadsheets. The Chi-square test was used to calculate the association between categorical variables as well as Phi to determine the level of association. Two group comparisons were made using the Mann-Whitney U test to assess whether the distribution of ranks was statistically significant. Odds ratio (OR) was used to determine the strength of association between bone mass and audiovestibular impairment symptoms, p-values < 0.05 were regarded as statistically significant.

## Results

Out of the 101 patients (median age=74 years; IQR=12), 56 were diagnosed with osteoporosis and 42 with osteopenia. No patient referred history of cerumen impaction, Meniere's Disease, myringoplasty, myringotomy, tympanoplasty, otosclerosis, or recurrent otitis media. Eight out of 11 patients with a family history of hearing loss were diagnosed with osteoporosis. One patient had cochlear implant history. 

Serum 25-(OH) vitamin D, corrected calcium, phosphate, albumin, and alkaline phosphatase demonstrated no significant difference between the means of any of the three groups (Table 1). Women with a history of vertigo revealed a significant association between the femoral neck T-scores and the FRAX scores, while women with a history of hearing difficulty showed significant association with both femoral neck and hip T-scores, as well as the FRAX scores. Women with a history of tinnitus showed no statistical significance with those previously mentioned. The BMI results exhibited no statistical significance for either of the three groups (Table 2).

**Table 1:** Laboratory tests associated with audiovestibular symptoms W= Mann-Whitney U test, NS= Not Significant

	History of Vertigo (n=26)			History of Hearing Difficulty (n=39)			History of Tinnitus (n=5)		
	Total	W	*p*-Value	Total	W	*p*-Value	Total	W	*p*-Value
25-(OH) Vitamin D (mg/dL) [Mean(SD)]	23.4(±8.2)	NS	0.440	24(±7.2)	NS	0.683	19.9(±6.2)	NS	0.194
Corrected Calcium (mmol/L)[Mean(SD)]	2.4(±0.1)	NS	0.332	2.4(±0.1)	NS	0.782	2.4(±0.1)	NS	0.944
Phospate (mmol/L) [Mean(SD)]	1.1(±0.2)	NS	0.225	2.4 (±0.332)	NS	0.941	1.2(±0.2)	NS	0.820
Albumin (g/dL) [Mean(SD)]	3.8(±0.6)	NS	0.143	3.9(±0.7)	NS	0.158	4.1(±0.4)	NS	0.975
Alkaline Phosphatase (U/L) [Mean(SD)]	85.8 (±28.3)	NS	0.408	86.4(±24.7)	NS	0.608	83.4(±17.9)	NS	0.826

**Table 2:** Scores associated with audiovestibular symptoms W: Mann-Whitney U test, NS: Not significant, FRAX: Fracture Risk Assessment Tool, BMI: Body mass index

	History of Vertigo (n=26)			History of Hearing Difficulty (n=39)			History of Tinnitus (n=5)		
	Total	W	p-value	Total	W	p-value	Total	W	p-value
Femoral Neck T-score [Mean(SD)]	-2.8 (±0.4)	585	0.002	-2.8 (±0.4)	804	0.005	-2.9 (±0.2)	NS	0.116
Hip T-score [Mean(SD)]	-2.7 (±0.5)	NS	0.132	-2.7 (±0.5)	894	0.028	-2.6 (±0.5)	NS	0.814
FRAX score [Mean(SD)]	25 (±3.6)	1384	0.001	22 (±25)	1610	0.004	24.5 (±2.2)	NS	0.317
BMI [Median(IQR)] (kg/ml²)	25.2 (4.5)	NS	0.302	25.4 (5.3)	NS	0.460	23.7 (3)	NS	0.476

Women with osteoporosis diagnosed using the femoral neck T-score demonstrated a greater level of association as well as higher odds of developing both vertigo and hearing difficulty than those diagnosed using the hip T-score. Women with a history of tinnitus had a statistically significant association with osteoporosis diagnosed using the femoral neck T-score (Table 3).

**Table 3:** Qualitative variables associated with audiovestibular symptoms NS: Not significant

	History of Vertigo (n=26)				History of Hearing Difficulty (n=39)				History of Tinnitus (n=5)			
	Total	Phi	*p*-Value	Odds Ratio	Total	Phi	p-value	Odds Ratio	Total	Phi	p-value	Odds Ratio
Osteoporosis by Femoral Neck T-score (n=56)	24	0.437	<0.001	2.780	31	0.384	<0.001	1.747	5	0.205	0.040	NS
Osteoporosis by Hip T-score (n=56)	19	0.209	0.036	1.025	28	0.261	0.009	1.128	4	NS	0.257	NS
Family History of Audiological Impairment (n=11)	4	NS	0.393	NS	6	NS	0.250	NS	1	NS	0.502	NS
Smoking History (n=13)	1	NS	0.111	NS	5	NS	0.990	NS	0	NS	0.378	NS

## Discussion

Due to inconsistencies in previous research, we decided to conduct the first nationwide study to investigate the relationship between bone mineral density and audiovestibular symptoms. We found, as well as other publications [2,4,8-9], a significant association in hearing impairment, vertigo, and tinnitus with the presence of osteoporosis. 

Presbycusis has been described to begin around the age of 60 as a symmetrical, slowly developing, high-frequency sensorineural hearing loss (SNHL) [2]. Identifying its precise cause has proven to be more difficult than initially thought, and as we have previously stated, many factors, including genetic and environmental, may be involved [1-4]. Furthermore, demineralized petrous temporal bone and age-related bone mass loss may contribute to its development [2].

Numerous studies on self-reported hearing loss have been conducted, where contradictory results have been identified. However, it has been proven to be strongly predictable at higher frequencies (4 to 8 kHz) hearing loss and in individuals aged 60 or older [12-13]. For the previously stated, we regarded self-reported hearing loss as a valid and useful tool.

As mentioned in earlier research [4,14], we discovered statistically significant higher odds of developing vertigo and hearing impairment in patients with a low femoral neck BMD, 1.7 more times of developing vertigo, and 2.9 more times of developing a hearing impairment. Increased odds of the aforesaid were also established in patients with low hip BMD.

As BMD of the femoral neck revealed a stronger association and OR with audiovestibular symptoms than the hip BMD, it's suggested that the femoral neck T-score could work as a more powerful predictor of hearing loss and vertigo development within these patients. This has been postulated in the past as a relation between the measured bone mass of the femoral neck and the petrous temporal bone because both contain a greater proportion of trabecular bone [14]. It has also been suggested, for the same reasons, that vertebral BMD could work as a better predictor than femoral neck and hip BMD [2]. However, this study did not include vertebral DXA, and further studies regarding this manner should be conducted. 

The present study must be viewed in light of its limitations. Hearing impairment outcomes were just determined by the use of a self-reported setting, for which it may not apply to other circumstances or populations. Our findings suggest that reduced BMD may be a risk factor for hearing impairment in postmenopausal women; therefore, it is essential to accurately identify osteoporotic patients with hearing loss and provide them with the proper follow-up.

## Conclusions

This study found a strong association between audiovestibular symptoms and osteoporosis in postmenopausal women. Therefore, we suggest paying special attention to the otologic evaluation of these patients, as well as increasing the referral to a specialized consult. 

Additional multicenter prospective national and regional studies are needed to complement these findings and better understand the specific patterns of audiovestibular disorders among these patients.
